# Building a large gene expression-cancer knowledge base with limited human annotations

**DOI:** 10.1093/database/baad061

**Published:** 2023-09-27

**Authors:** Stefano Marchesin, Laura Menotti, Fabio Giachelle, Gianmaria Silvello, Omar Alonso

**Affiliations:** Department of Information Engineering, University of Padova, Via G. Gradenigo 6b, Padova 35131, Italy; Department of Information Engineering, University of Padova, Via G. Gradenigo 6b, Padova 35131, Italy; Department of Information Engineering, University of Padova, Via G. Gradenigo 6b, Padova 35131, Italy; Department of Information Engineering, University of Padova, Via G. Gradenigo 6b, Padova 35131, Italy; Applied Science, Amazon, 3075 Olcott St., Santa Clara, California 95054, USA

## Abstract

Cancer prevention is one of the most pressing challenges that public health needs to face. In this regard, data-driven research is central to assist medical solutions targeting cancer. To fully harness the power of data-driven research, it is imperative to have well-organized machine-readable facts into a knowledge base (KB). Motivated by this urgent need, we introduce the Collaborative Oriented Relation Extraction (CORE) system for building KBs with limited manual annotations. CORE is based on the combination of distant supervision and active learning paradigms and offers a seamless, transparent, modular architecture equipped for large-scale processing. We focus on precision medicine and build the largest KB on ‘fine-grained’ gene expression–cancer associations—a key to complement and validate experimental data for cancer research. We show the robustness of CORE and discuss the usefulness of the provided KB.

**Database URL** https://zenodo.org/record/7577127

## Introduction

In 2020, there were 19.2 million cancer cases worldwide. The World Health Organization estimates a 32% overall increase by 2040.^1^ With this growing global burden, cancer prevention is one of the century’s most pressing public health challenges and data-driven research is crucial for assisting the development of medical solutions to address them. In the last few years, cancer research increasingly relied on microarray and next-generation sequencing technologies, which provide raw ‘experimental data’ about ‘gene expression–cancer interactions’ (1, 2). These interactions hold vital information to guide diagnosis, assess prognosis or predict therapy response (3, 4). To fully harness the potential of this data, they must be made readily accessible and organized in a comprehensive manner. This is where Knowledge Bases (KBs) come into play, providing machine-readable knowledge that connects cutting-edge technologies with data-driven research to support the fight against cancer (5).

Although experimental data are invaluable for cancer research, they require further investigation, processing and validation by experts to be used to populate a KB. Luckily, in most cases, the analysis and interpretation of experimental data are described in peer-reviewed publications, making the scientific literature a critical source to complement and validate the data. Given the high economical and time costs to manually extract knowledge ( e.g. scientific facts) from the domain-specific literature (6–8), in recent years, Machine Learning (ML) methods and automated techniques for Knowledge Base Construction (KBC) have flourished (9–11). The main bottleneck for KBC systems is the requisite of large and expensive labeled training data to perform Named Entity Recognition and Disambiguation () and Relation Extraction (RE).

Distant supervision (12, 13) and active learning (14, 15) are the main paradigms adopted to address this limitation (16, 17). In this work, we use both paradigms in conjunction to build a modular, pluggable, transparent and scalable KBC system focusing on the discovery of ‘gene expression–cancer’ associations.

We note that there are a handful of knowledge resources containing data about gene expression–cancer associations (18–23) and most of them only contain experimental data (18–21). A small minority—e.g. BioXpress (22) and OncoMX (23)—also integrates knowledge extracted from the biomedical literature. To do so, they rely on Disease-Expression Relation Extraction from Text (DEXTER) (24), a state-of-the-art method based on ‘pattern matching’ techniques. CoMAGC (25) and OncoSearch (26) are other literature-based resources (27, 28), modeling also the particularly valuable fine-grained aspects of gene expression–cancer associations. Still, CoMAGC only consists of 821 sentences on prostate, breast and ovarian cancers, while OncoSearch is not maintained.

More general and large-scale resources on gene–disease associations—i.e. DisGeNET (27) and the Literature-derived Human Gene-Disease Network (LHGDN) (28)—store coarse-grained information expressing the existence of an association between gene expression and cancer, which is often insufficient to model such complex, faceted relationships effectively.

Hence, there is a need for KBC systems that can scale to large text corpora and stay up to date while generating fine-grained information about gene expression–cancer associations. We present the Collaborative Oriented Relation Extraction (CORE) system, a KBC system based on the combination of automated ML-based methods and domain experts’ feedback. CORE features a seamless, transparent, modular architecture, where the different components can be easily plugged-in. It also employs active learning to bootstrap the KB to be produced and exploits the fine-grained aspects involved in gene expression–cancer associations to perform iterative tests that measure the reliability of the data to be stored in the KB. Finally, it returns small, selected samples to domain experts for annotation. The high-quality data generated by this process are then used as reinforcement to retrain the ML models from scratch. Active learning makes CORE suited to iterative KB versioning. Therefore, with the data annotated by domain experts, retrained ML models are deployed to build subsequent versions of the KB.

The experimental evaluation shows the robustness of the proposed approach by highlighting how CORE scales to large text corpora with little human annotations. Additionally, when compared to the state-of-the-art in a KB reconstruction task, the performance of CORE confirms the system effectiveness in this domain. We have made the KB derived by CORE available as Open Data (29), provided with a SPARQL Protocol and RDF Query Language (SPARQL) endpoint for querying: http://w3id.org/corekb/sparql. The KB can also be accessed via COREKB (30), an intuitive and easy-to-use search platform: https://gda.dei.unipd.it. The source code is available at https://github.com/GDAMining/core.

The rest of the article is organized as follows: Section (Background) presents the required background; Section (The CORE system) outlines the CORE system; Sections (NERD)–(Active learning) describe the components of CORE; Section (Implementation and experiments) presents the system settings and the experiments; Section (KB exploration) performs some exploratory queries and analyses on the generated KB; Section (Search platform) showcases the search platform and Section (Conclusions and future work) draws final remarks.

## Background

### Precision medicine and gene expression

Generally, precision medicine is about tailoring of medical treatment to the characteristics of an individual patient and moves beyond the traditional approach of stratifying patients into treatment groups based on phenotypic markers (31). Among the different fields of medicine, precision medicine has the greatest impact in cancer research. In this context, precision medicine involves the use of an individual patient’s genomics information to guide diagnosis, prognosis, treatment and prevention of cancer for that patient. In other words, precision medicine is a multifaceted approach that involves several critical aspects such as pharmacogenomics, pharmacodynamics and the impact of genetic variations on an individual’s response to cancer treatments like chemotherapeutics (32). It also considers various factors, including age, ethnicity, sex and lifestyle habits, as prognostic indicators, as well as diagnostic aspects related to disease identification and its severity. Among the factors that identify the different risk outcomes in patients, alterations in gene expression patterns play a central role (3). In fact, genes contain the information required to create proteins and dictate cellular functions, but it is the ‘gene expression’ that determines the cellular phenotype—and therefore the disease development. Moreover, abnormalities in the expression of microRNAs—small RNAs that post-transcriptionally regulate the expression of their target genes—have recently been associated with cancer (33, 34). Thus, identifying genes and microRNAs whose expression levels interact with cancer status is imperative to advance cancer research (3).

### Gene expression–cancer resources

Most knowledge resources about ‘gene expression–cancer associations’ consist of experimental data obtained through microarray and next-generation sequencing technologies (18–21). A relevant example is GENT2 (18), which provides a search platform for gene expression patterns across different normal and tumor tissues, compiled from public gene expression datasets. Another platform is the Metabolic gEne RApid Visualizer (19), which provides access to gene expression datasets and compares gene expressions across human tissues and cell types. The International Cancer Genome Consortium (20) is a collaborative effort to characterize genomic abnormalities in 50 different cancer types, which provides a data portal containing data from 24 cancer projects, including The Cancer Genome Atlas (21). As explained earlier, these resources are valuable but provide raw data that need to be further processed and validated to be effectively used in precision medicine.

Beyond experimental data, BioXpress (22) and OncoMX (23) also integrate knowledge extracted—either manually or automatically—from the biomedical literature (22, 23). BioXpress is a KB storing genes that are differentially expressed in adjacent normal and tumor tissues from the same patients. On the other hand, OncoMX is a KB for exploring cancer biomarkers, which encompasses more than 1000 unique biomarker entries mapped to 20 576 genes that have either mutations or differential expressions in cancer. In particular, both resources consider data automatically extracted by DEXTER (24), a text-mining expert system that identifies gene and microRNA expressions in disease samples from sentences selected from the relevant literature. To the best of our knowledge, DEXTER is the most advanced text-mining system for gene expression–cancer associations. However, the use of pattern matching techniques—based on manually defined regular expressions—to extract relationships limits DEXTER flexibility and hampers its applicability to a broad range of new, unseen sentences. In this regard, we show that DEXTER fails to extract knowledge from sentences whose syntactic structure differs from its predefined patterns. Thus, it appears clear that more adaptive RE methods are required to build KBC systems capable of scaling to large-scale text corpora, made of heterogeneous documents written in different styles.

Regarding literature-based resources, CoMAGC (25) is a corpus developed to train RE methods targeting gene expression–cancer associations. In CoMAGC, each sentence is annotated with different concepts that together express (i) how a gene changes, (ii) how a cancer changes and (iii) the interaction between gene and cancer changes. Together, the different concepts can be used to infer the prospective roles of genes in cancer and to classify genes into classes according to the inferred roles. Relying on the CoMAGC annotation schema, OncoSearch (26) retrieves sentences mentioning gene expression changes in cancers from Medline abstracts. Specifically, OncoSearch queries seek (i) whether a gene is up- or downregulated, (ii) whether a given cancer progresses or regresses based on the given gene expression change and (iii) the expected role of the gene in the cancer. While relevant, CoMAGC is small-scale and OncoSearch is not maintained, making them limited resources. On the other hand, general, large-scale and widely used resources about gene–disease associations, such as DisGeNET (27) and LHGDN (28), store information that is not specific enough to be effectively used to model gene expression–cancer relationships.

### Knowledge base construction

KBs have gained a great deal of attention recently as a key component for supporting search and recommendations at the Web scale. From seminal works such as DBPedia (35), Freebase (36) and YAGO (37) to community-driven projects such as WikiData (38), KBs have become a central asset in several applications. To build them, KBC spans different areas of data management and artificial intelligence. Data integration (39, 40), data cleaning (41), NERD (42–44), RE (45–47) and active learning (14, 15, 48) are critical to ensure the accurate and scalable construction of KBs. In this regard, several systems that include the latest advances in these areas have been built  (9–11, 49).

KBs are also increasingly used by large companies and organizations as a means for organizing and understanding their data (50–53). Industry-scale KBs are typically derived from numerous sources and contain a wealth of information that is used in downstream applications (54). One of the few published reports of an end-to-end industrial system to build, maintain and use such KBs is the work by Deshpande et al. (50). We follow a similar focus on data quality and clear methodology for bootstrapping a KB. Besides, we agree that an imperfect KB is still useful for real-world applications and that maintenance is also important as facts evolve over time.

## The CORE system

### Preliminaries

Let us consider a directed graph $G=(V,E)$, where $E \subseteq \{(v_1,v_2)\ |\ (v_1,v_2)\in V \times V\}$ is the set of edges connecting ordered pairs of vertices. Given an edge $e = (v_1,v_2) \in E$, we call *v*_1_ the source vertex and *v*_2_ the target vertex. In our context, the nodes of *G* are entities and the edges are the relationships between them.

Definition 1. **Aspect**

We call aspect an attribute of a relationship between a pair of entities. An aspect *A*_*i*_ has a name and a domain ${D}=\{a_{i1}, \ldots, a_{in}\}$, where $a_{ij}\in A_i$ is the *j*^*th*^*aspect value* of *A*_*i*_. $Dom(A_i) = {D}$ returns the domain of *A*_*i*_ (when it is clear from the context, the aspect value $a_{ij} \in A_i$ is referred to as *a*_*j*_).

Example 1. Let us consider gene–cancer associations. There are three aspects describing a possible relationship (*e*) between gene (*v*_1_) and cancer (*v*_2_): the change of gene expression (CGE), the change of cancer status (CCS) and the gene–cancer interaction (GCI). Following Definition 1, CGE, CCS and GCI are the names of the aspects with the following domains: $Dom(\text{CGE}) = \{\texttt{up}, \texttt{down}, \texttt{notinf}\}$, $Dom(\text{CCS}) = \{\texttt{progression}, \texttt{regression}, \texttt{notinf} \}$ and *Dom*(GCI) = {causality, correlation, notinf}.

Details about the aspect domains are given in Table 1.

**Table 1.** Description of the aspects involved in gene expression-cancer associations. For each aspect, we report its domain values and the corresponding descriptions.

Aspect	Value	Description
CGE	up	The expression of a gene is increased.
	down	The expression of a gene is decreased.
	notinf	The change of gene expression is unknown.
Change of cancer status (CCS)	progression	The cell or tissue acquires cancerous properties as gene expression level changes.
	regression	The cell or tissue loses cancerous properties as gene expression level changes.
	notinf	The change of cancerous properties of cell or tissue is unknown.
Gene–cancer interaction (GCI)	causality	There is a cause-effect relationship between CGE and CCS.
	correlation	There is a correlation between CGE and CCS.
	notinf	The interaction between CGE and CCS is unknown.

Definition 2. **multi-aspect relationship**

Given a graph $G(V,E)$ and a set of aspects $\mathcal{A} = \{A_i\}_{i=1}^{n}$, a tuple of aspect values $(a_{1j}, \ldots, a_{nj})$ associated with $e = (v_1,v_2)$$\in E$ defines a multi-aspect relationship between *v*_1_ and *v*_2_.

Definition 3. **signature function**

Given a set of aspects $\mathcal{A} = \{A_i\}_{i=1}^{n}$ and an alphabet Σ, we define $\operatorname{s}: \prod_{i=1}^{n}A_i \rightarrow S \subseteq \Sigma^{*}; \operatorname{s}(a_{1j}, {\ldots}, a_{nj}) \mapsto \texttt{type}$ as the signature function that maps a multi-aspect relationship to a type in *S*, called the signature set.

The signature function defines a set of mapping rules depending on the domain of interest. We use the signature function to map multi-aspect gene expression–cancer relationships to gene prospective roles in cancer ( e.g. oncogene or biomarker). Table 2 provides the inference rules used to derive the expected gene roles. Gene roles allow us to distinguish the genes that are responsible for oncogenesis from those that are not; this is essential information for effective cancer research and therapy design (55).

**Table 2.** Inference rules for gene classes. For each combination of CGE, CCS and GCI, we report the expected gene class. Gene classes refer to the role that a given gene plays in a specific disease. Following (25, 26), a biomarker represents a gene that exhibits altered expression levels in cancer, but which is not (yet) identified as an oncogene or a tumor suppressor gene. In Rule 5, CGE and CCS can assume any value between $\{\texttt{up}, \texttt{down} \}$ and $\{\texttt{progression}, \texttt{regression}\}$.

Rule number	CGE	CCS	GCI	Gene Class
1	up	progression	causality	oncogene
2	up	regression	causality	tumor suppressor gene
3	down	regression	causality	oncogene
4	down	progression	causality	tumor suppressor gene
5	up|down	progression|regression	observation	biomarker

Definition 4. **tagging function**

Given an edge $e \in E$ and the signature set *S*, we define $\sigma:E \rightarrow S; \sigma(e) \mapsto $type as the function tagging an edge with a signature type.

The tagging function associates a signature type with an edge of the graph. In this work, we use the tagging function to label edges with gene prospective roles.The graph represents gene expression–cancer associations as gene prospective roles in cancer.

### Overview

The goal of the CORE system is to harvest facts from text corpora to populate KBs. We model a KB as a directed graph *G* made up of entities connected by typed relationships. Facts (or statements) are $(v_{1}, e, v_{2})$ triples, where $v_{1}, v_{2} \in V$, $e = (v_1,v_2) \in E$ and $\sigma(e) \in S$.

To obtain facts, CORE collects the scientific literature from different sources, identifies sentences containing pairs of entities relevant to the considered task and extracts aspects from them. Depending on the combination of extracted aspect values, a sentence expresses a specific signature type. Note that, for a given pair of entities, different sentences can express various signature types, as we show in the next example.

Example 2. See these two sentences from the biomedical literature:

- ***Colorectal cancer** (CRC) growth and progression is frequently driven by RAS pathway activation through upstream growth factor receptor activation or through mutational activation of KRAS or **BRAF**.*
- *Somatic mutations of the BRAF gene, causing constitutive activation of **BRAF**, have been found in various types of human cancers such as malignant melanoma, and **CRC**.*

In both sentences, the following entities are extracted $v_1 = \texttt{BRAF}$ and $v_2 = \texttt{CRC}$. Considering the aspects introduced in Example 1, for sentence A, we find CGE = up, CCS = progression and GCI = causality, leading to the signature type s((up, progression, causality)) = oncogene. On the other hand, the aspect values of sentence B are CGE = up, CCS = progression and GCI = correlation, leading to the signature type s((up, progression, correlation)) = biomarker.

From Example 2, we see that different sentences may lead to different signature types. In the scientific discourse, it is not surprising that there are different viewpoints and that various studies can lead to different conclusions—even in contradiction with each other. Hence, we need to consider this potential uncertainty when facts are extracted from the literature. The CORE system models this inherent uncertainty by assigning the likelihood of being true to each aspect value. This probability is based on the evidence we can extract from the literature. Given a set of sentences concerning the same two entities, the more an aspect value is consistent in the set, the higher the probability for that value to be true.

Definition 5. **aspect–probability set**

Given an aspect $A_i = \{a_j\}_{j=1}^{m}$ such that each aspect value *a*_*j*_ carries a likelihood $\operatorname{Pr}(a_{j})$, we call $AP_{i} = \{(a_{j}, \operatorname{Pr}(a_{j}))\}_{j = 1}^{m}$ the aspect–probability set of *A*_*i*_.

Definition 6. **multi-aspect function**

Let $G = (V,E)$ be a directed graph and $\mathcal{AP} = \{AP_i\}_{i=1}^{n}$ a set of aspect–probability sets. We define $\phi: E \rightarrow \prod_{i=1}^{n}AP_{i}; \phi(e) \mapsto (\{(a_{1j}, \operatorname{Pr}(a_{1j}))\}_{j=1}^{|A_{1}|}, \dots, \{(a_{nj}, \operatorname{Pr}(a_{nj}))\}_{j=1}^{|A_{n}|})$ as the multi-aspect function that, given an edge, returns the *n*-tuple of aspect–probability sets.

Thus, for each pair of target entities, CORE computes the probabilities for all the aspect values and combines them into tuples of aspect–probability sets—i.e. a probability distribution over multi-aspect relationships. Sentences serve as supporting or contradicting evidence that strengthens or weakens the likelihood of a fact.

### Architecture

**Figure 1.**
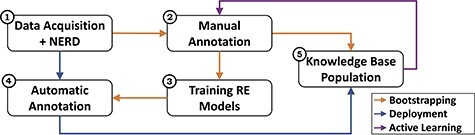
Overview of the CORE architecture. The system consists of five main modules and three processes. The modules represent the data acquisition and NERD components (1), the manual annotation activities (2), the training of the RE models (3), the subsequent automatic annotation (4), and the KB population (5). The processes reflect the different workflows: bootstrapping (orange) sets up the KBC process via expert involvement; deployment (blue) scales it through automated RE methods; and active learning (purple) allows refining the process through subsequent iterations.

**Figure 2.**
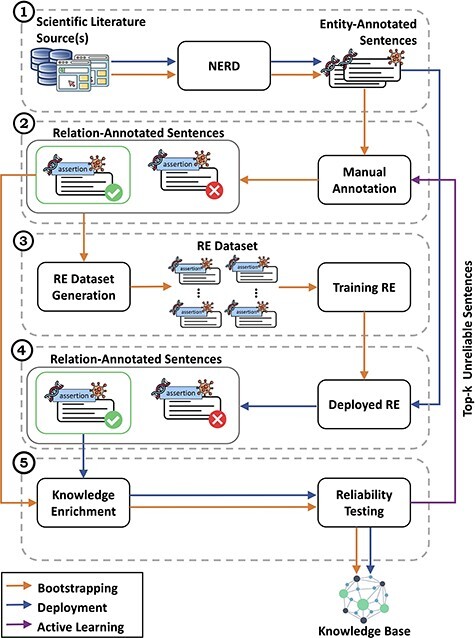
Detailed view of the CORE architecture. In module (1), CORE acquires text from biomedical literature and then performs NERD to generate entity-annotated sentences. These sentences are then manually annotated by experts in module (2) to produce relation-annotated sentences, which are used to generate the datasets for training RE methods in module (3). Once trained, in module (4), the RE methods are deployed over entity-annotated sentences to automatically generate relation-annotated sentences. Finally, in module (5), relation-annotated sentences undergo a knowledge enrichment component, which generates facts, and a reliability testing component, which tags facts as ‘reliable’ or ‘unreliable’. Facts tagged as *‘reliable’* are used to populate the KB, whereas *‘unreliable’* facts are returned to experts for re-annotation.

Figure 1 gives an overview of the CORE architecture, depicting modules and processes, and Figure 2 zooms into it providing further details. The system acquires text from the literature and processes and normalizes it to obtain sentences, from which a NERD component detects and annotates the entity pairs (Module 1). These entity-annotated sentences undergo two different processes: bootstrapping and deployment. In the bootstrapping workflow, experts manually annotate multi-aspect relationships between the entities (Module 2), producing a set of ‘relation-annotated sentences’ used to train RE methods (Module 3) and to populate the KB (Module 5).

In the deployment workflow, the automatic annotations expressing multi-aspect relationships between entities are provided by the RE methods (Module 4) previously trained in the bootstrapping phase. Then, in the last module (Module 5), relation-annotated sentences are grouped by entity pairs and used to generate facts to further populate the KB. Module 5 is composed of (i) a knowledge enrichment component computing the probabilities for all the aspect values and combining them into tuples of aspect–probability sets and (ii) a reliability testing component that uses these probabilities to perform multiple tests to tag the facts as ‘reliable’ or ‘unreliable’. Only the facts tagged as ‘reliable’ are used to populate the KB. When the deployment workflow is complete, ‘unreliable’ facts are ranked by ascending reliability score and the top-*k* automatically annotated sentences associated with them are reannotated by experts. This process triggers an active learning workflow that reinforces the RE methods.

### Versioning

The active learning workflow makes CORE suitable to iterative KB versioning. We define a KB version as the graph $G_{j} = (V_{j}, E_{j})$ obtained after the *j*^*th*^ iteration of the bootstrap and deployment workflows. Once the *j*^*th*^ version of the KB has been deployed, the active learning workflow starts by generating the batch of unreliable sentences for bootstrapping the $j^{th}+1$ version of the KB. The unreliable sentences are manually annotated and used to increase the size of the datasets to retrain the RE methods from scratch, which then generate a new set of automatic annotations to be included in the $j^{th}+1$ KB version. Hence, once the bootstrap and deployment workflows are completed, the $j^{th}+1$ version of the KB is rebuilt from scratch and comprises all the available annotations.

## NERD

CORE recognizes gene and cancer entities from text and links them to relevant and authoritative KBs. In our setting, gene entities are linked to the National Center for Biotechnology Information (NCBI) Gene database (56), whereas cancer entities are linked to the Unified Medical Language System (UMLS) (57). The choice of UMLS as the reference KB for cancer aims to maximize the interoperability of the CORE system with different existing biomedical resources, such as DisGeNET, BioXpress and OncoMX.

As NERD component, CORE integrates the PubTator system (58–60). Given biomedical text, PubTator provides automated annotations from state-of-the-art text mining systems for genes/proteins, genetic variants, diseases, chemicals, species and cell lines. In particular, PubTator normalizes annotated genes to NCBI Gene identifiers and annotated diseases to MeSH (61) identifiers. However, the CORE system requires UMLS identifiers for diseases. Therefore, a mapping process normalizes MeSH identifiers to UMLS Concept Unique Identifiers (CUIs). Then, to restrict to cancer, CORE only keeps UMLS CUIs that belong to the neoplastic process’ semantic type. Once gene and cancer entities have been extracted and linked to the reference KBs, CORE splits biomedical text into sentences and keeps only those sentences containing gene–cancer pairs. When a sentence contains multiple gene–cancer pairs, CORE returns separate entity-annotated sentences for each pair.

## Manual annotation

CORE involves experts to manually annotate some sentences with multi-aspect relationships about gene expression–cancer associations. For annotation, CORE adopts a common and shared schema in the biomedical domain (e.g. CoMAGC (25) and OncoSearch (26)), where experts are required to annotate sentences with three different aspects: CGE, CCS and GCI. CGE represents the change of the gene expression level, CCS represents the change of the cancer status and GCI indicates the interaction occurring between CGE and CCS aspects. Each aspect can assume different values: $Dom(\text{CGE}) = \{\texttt{up}, \texttt{down}, \texttt{notinf}\}$; *Dom*(CCS) = {progression, regression, notinf}; *Dom*(GCI) = {causality, correlation, notinf}.

Even though a huge amount of sentences contains gene–cancer pairs in the biomedical literature, only a small fraction actually describes gene expression–cancer associations. It is therefore essential to have a tool capable of limiting the amount of noise introduced in the annotation process and maximizing sentence utility. In this regard, CORE requires the annotation of an additional aspect, the gene–cancer context (GCC). GCC indicates the coarse-grained association between gene and cancer and serves as a filter that helps differentiating between gene–cancer associations related to changes in the gene expression levels and those encompassing other types of gene–cancer relationships. To this end, GCC has the following domain: $Dom(\text{GCC}) = \{\texttt{expression}, \texttt{other}\}$. The $\texttt{expression}$ value indicates that an altered gene expression is associated with cancer, whereas the $\texttt{other}$ value represents any other gene–cancer association(note that $\texttt{other}$ can be broken down into different and finer values, thus leaving room for the integration of different types of gene–cancer associations in the CORE system.)—including the absence of association. Thus, the GCC aspect assesses the sentence utility in context, because it is a filter limiting manual and automatic analysis of sentences containing gene–cancer pairs not inherent to gene expression–cancer associations.

Based on the annotation schema presented earlier, domain experts perform multi-aspect manual annotations between gene–cancer pairs and return relation-annotated sentences (Module 2). Depending on the considered workflow, the sentences to be annotated come from different modules at different stages. At the beginning of bootstrapping, (entity-annotated) sentences come from Module 1 as the output of the NERD component. After active learning, (unreliable) sentences come from Module 5 due to the reliability testing. In both cases, any errors associated with the NERD component are corrected too. For training RE methods (Module 3), the CORE system employs the complete set of relation-annotated sentences, whereas to populate the KB (Module 5), CORE keeps only the sentences with $\text{GCC} =\texttt{expression}$. The sentences annotated with $\text{CGE} = \texttt{notinf}$ are excluded to limit noise injection, because CGE is the main aspect gene expression–cancer associations driver (25).

## Relation extraction

CORE’s RE methods are trained to automatically annotate multi-aspect relationships on sentences. Once trained, the RE methods are applied on new, unseen sentences to generate knowledge and thus update the KB.

For each aspect to be annotated, a different RE method is trained (Module 3) and deployed (Module 4). Together, different aspect-based annotations compose the multi-aspect relationship. Although simple, this approach reflects the transparent and modular architecture of the CORE system, where different components can be easily plugged in and plugged out since every RE method can be retrained—or changed—without affecting others.

The RE methods serve two purposes: classify sentence utility and extract gene expression–cancer aspects. Every RE method presents the same architecture but addresses a different aspect. As the underlying ML model, all RE methods adopt SciBERT (62), a pretrained language model based on BERT (63). SciBERT addresses the lack of high-quality, large-scale labeled scientific data by pretraining on scientific papers from Semantic Scholar (64). On top of it, a linear layer takes SciBERT pooled output. Predictions are scores in $[0,1]$ for target values. The higher the score for an aspect value, the more the RE method believes the sentence expresses that particular value. We formally define prediction scores as follows.

Definition 7. **score function**

Let *A*_*i*_ be an aspect, *a*_*j*_ one of its values and *T* a set of sentences. We define $\operatorname{score}: A_{i} \times T \rightarrow \mathbb{R}_{[0,1]}; \, \operatorname{score}(a_{j}, t) \mapsto r$ as the score function that given a sentence *t* returns how close the aspect value *a*_*j*_ is to the truth.

Every RE method instantiates the score function tailored for its aspect extraction task. A RE method returns a specific sentence-aspect score always in the range of $[0,1]$ of real numbers.

Remark 1. Given a sentence *t* and an aspect $A_{i} = \{a_{j}\}_{j=1}^{m}$, then $\sum_{a_{j} \in A_{i}} \operatorname{score}(a_{j}, t) = 1$. Prediction scores for an aspect *A*_*i*_ given a sentence *t* are a probability distribution over the aspect values *a*_*j*_.

Thus, given an entity-annotated sentence (Module 1), CORE first masks gene and cancer entities with special tokens to avoid bias and then applies RE methods to extract CGE, CCS, GCI and GCC aspects. GCC extraction serves to assess sentence utility, while CGE, CCS and GCI extraction to compose multi-aspect relationships. For each aspect, the CORE system keeps the value associated with the highest score. For manual annotation, the scores are set to 1 if the aspect value is present and 0 otherwise.

Afterward, relation-annotated sentences where the extracted GCC value is $\texttt{expression}$ are kept, whereas those with $\text{GCC} = \texttt{other}$ are discarded. As in manual annotation, sentences with $\text{CGE} = \texttt{notinf}$ are also filtered out. The retained set of automatic, relation-annotated sentences is used to populate the KB (Module 5).

## Knowledge enrichment

The relation-annotated sentences obtained from manual annotation (Module 2) and RE deployment (Module 4) pass through a knowledge enrichment component, which groups annotated sentences by gene–cancer pairs and generates facts. However, for a given gene–cancer pair, different sentences can have different multi-aspect annotations. This situation occurs because, in the literature, different studies and viewpoints can lead to different conclusions. To address this intrinsic uncertainty, the CORE system assigns to each aspect a likelihood to be true.

Definition 8. **aspect value likelihood**

Let $(v_{1}, v_{2})$ be a pair of entities, $T_{(v_{1}, v_{2})}$ the set of sentences annotated with both *v*_1_ and *v*_2_ and $a_{j} \in A_{i}$ the target aspect value. Then, the aspect value likelihood is

(1) $$ \operatorname{Pr}(A_{i}=a_{j}\mid(v_{1}, v_{2})) = \frac{\sum_{t \in T_{(v_{1}, v_{2})}} \text{score}(a_{j}, t) \cdot \mathbb{1}(a_{j}, t)}{\sum_{t \in T_{(v_{1}, v_{2})}} \max\limits_{a_{k} \in A_{i}}(\text{score}(a_{k}, t))}, $$

where $\mathbb{1}(\cdot, \cdot)$ represents the indicator function, defined as


(2) $$ \!\!\!\!\!\!\!\!\!\mathbb{1}(a_{j}, t) = \begin{cases} \frac{1}{|\operatorname{arg\,max}\limits_{a_{k} \in A_{i}}(\text{score}(a_{k}, t))|}, & a_{j} \in \operatorname{arg\,max}\limits_{a_{k} \in A_{i}}(\text{score}(a_{k}, t)) \\ 0, & \text{otherwise}. \end{cases} \\[-28pt]\nonumber$$

By modeling aspect value likelihoods this way, the CORE system takes the beliefs of RE methods into account. The more the RE methods are confident about an aspect value *a*_*j*_ over the others, the more its likelihood increases. Vice versa, if RE methods have a larger degree of uncertainty across the different aspect values, then the likelihood for *a*_*j*_ also decreases accordingly.

Remark 2. Given a pair of entities $(v_{1}, v_{2})$, its set of annotated sentences $T_{(v_{1}, v_{2})}$ and an aspect *A*_*i*_, then $\sum_{a_{j} \in A_{i}} \operatorname{Pr}(A_{i} = a_{j}\mid(v_{1}, v_{2})) = 1$.

Example 3. Let us consider a gene–disease pair $(v_{1}, v_{2})$, its set of annotated sentences $T_{(v_{1}, v_{2})} = \{t_{1}, t_{2}, t_{3}, t_{4} \}$ and the CGE, CCS and GCI aspects. For each sentence, the candidate aspect value–score pairs are as follows:

$t_{1}:$

: CGE (up, 0.7), CCS(progression, 0.6), GCI(notinf, 0.9)

$t_{2}:$

: CGE (down, 0.8), CCS (regression, 0.9), GCI (causality, 0.6),

$t_{3}:$

: CGE (notinf, 0.8), CCS (progression, 0.9), GCI(notinf, 0.9),

$t_{4}:$

: CGE (up, 1.0), CCS (regression, 1.0), GCI (observation, 1.0).

First, sentence *t*_3_ is filtered out since CGE = notinf. Hence, the sentences used for computation are *t*_1_, *t*_2_ and *t*_4_. Then, following Definition 8, CGE value likelihoods are computed as Pr(up) = (0.7 + 0.0 + 1.0)/(0.7 + 0.8 + 1.0) = 0.68 and Pr(down) =(0.0 + 0.8 + 0.0)/(0.7 + 0.8 + 1.0) = 0.32, leading to the aspect–probability set *AP*_CGE_ = {(up, 0.68), (down}, 0.32)}. Similarly, CCS and GCI lead to aspect–probability sets *AP*_CCS_ = {(progression, 0.24), (regression, 0.76), (notinf, 0.00)} and *AP*_GCI_ = {(observation, 0.40), (causality, 0.24), (notinf, 0.36)}. Thus, given the fact $(v_{1}, e, v_{2})$ obtained from the gene–disease pair $(v_{1}, v_{2})$, we have that $\phi(e) = (AP_{\text{CGE}}, AP_{\text{CCS}}, AP_{\text{GCI}})$ consists of the aspect–probability sets defined earlier.

For each fact, CORE combines CGE, CCS and GCI aspects into the tuple of aspect–probability sets, which represents a probability distribution over multi-aspect relationships and performs reliability tests to decide if the fact is reliable enough to populate the KB.

## Reliability testing

The facts generated through the knowledge enrichment component undergo a set of reliability tests, which are used by CORE to identify those facts that are reliable enough to populate the KB. These reliability tests are based on aspect–probability sets and follow the inference rules defined in (25, 26) and reported in Table 2 to map multi-aspect relationships to signature types. Indeed, multi-aspect relationships can be used to infer the prospective roles of genes in cancer and to classify genes into three mutually exclusive classes according to the inferred role: $\texttt{oncogene}$, $\texttt{tumor suppressor gene}$ and $\texttt{biomarker}$ (as in (25, 26), a gene classified as $\texttt{biomarker}$ represents a gene that exhibits altered expression levels in cancer, which, however, is not (yet) identified as $\texttt{oncogene}$ or $\texttt{tumor suppressor gene}$). For instance, an $ \texttt{oncogene}$ can be inferred from $( \texttt{up}, \texttt{progression}, \texttt{causality})$ or (down, regression, causality) multi-aspect relationships (Rules 1 and 3 of Table 2). These mutually exclusive classes represent the signature set *S* and are associated with edges of the KB through the tagging function $\sigma(\cdot)$.

Thus, based on aspect–probability sets and inference rules, CORE performs a two-stage reliability test that first verifies that facts have sufficient evidence and then assesses the degree of contradicting evidence. The two stages are divided into sufficiency and consistency checks.

Given a fact $(v_{1}, e, v_{2})$, a sufficiency check monitors whether the likelihood of not-informative aspect values is large enough to undermine the reliability of the fact. CORE applies the sufficiency check to $\text{CCS} = \texttt{notinf}$ and $\text{GCI} = \texttt{notinf}$ aspect values. Hence, a fact fails the sufficiency check and therefore is deemed ‘unreliable’ if $ \operatorname{Pr}(\text{CCS}= \texttt{notinf}) \gt \alpha \lor \operatorname{Pr}(\text{GCI}=\texttt{notinf}) \gt \alpha $, where $ \alpha$ is a fixed system threshold.

The facts that pass the sufficiency check are further inspected for consistency. Given a tuple of aspect–probability sets, associated with a fact $(v_{1}, e, v_{2})$ through $\phi(e)$, the consistency check verifies that mutually exclusive signature types are not similarly probable.

Definition 9. **signature type likelihood**

Let $(v_{1}, e, v_{2})$ be a fact and *S* the set of mutually exclusive signature types. Then, the signature type likelihood is defined as

(3) $$ \operatorname{Pr}(\sigma(e) = \texttt{type}) = \sum_{\substack{\{(a_{1j}, ..., a_{nj}) \, \text{s.t.} \\ \operatorname{s}((a_{1j}, ..., a_{nj})) = \texttt{type} \}}} \prod_{i=1}^{n} \operatorname{Pr}(a_{ij}), $$

where $\operatorname{Pr}(a_{ij})$ is the aspect value likelihood and $\sigma(\cdot)$ and $\operatorname{s}(\cdot)$ are the tagging and signature functions, respectively.

Since gene expression–cancer aspects can be treated as independent events (25, 26), the signature type likelihood can be computed for the gene classes. For instance, according to Rules 1 and 3 from Table 2, the likelihood of the $\texttt{oncogene}$ class is


\begin{equation*}\begin{split} \operatorname{Pr}(\texttt{oncogene}) = & \operatorname{Pr}(\texttt{up}) \cdot \operatorname{Pr}(\texttt{progression}) \cdot \\ &\operatorname{Pr}(\texttt{causality}) + \operatorname{Pr}(\texttt{down}) \cdot \\ &\operatorname{Pr}(\texttt{regression}) \cdot \operatorname{Pr}(\texttt{causality}). \end{split}\end{equation*}

Given that gene classes are mutually exclusive, the consistency check verifies whether the class likelihoods are too close to each other. Indeed, similar likelihoods imply that a fact is supported by contradictory evidence, thus showing some inconsistency. Vice versa, a large difference between likelihoods suggests a strong tendency towards a specific gene class, and therefore a more consistent support for the fact.

Hence, for a target fact $(v_{1}, e, v_{2})$, CORE takes gene classes $\texttt{type-1}$ and $\texttt{type-2}$ with largest likelihoods and verifies that the condition $ (\operatorname{Pr}(\texttt{type-1}) - \operatorname{Pr}(\texttt{type-2})) \gt \beta$ is satisfied, where $ \beta$ is a fixed system threshold. A fact that fails the condition is therefore considered ‘unreliable’. In other words, when no gene class has a likelihood large enough to overcome the others by a margin of *β*, CORE tags the fact as ‘unreliable’. Note that the consistency check admits that only one gene class satisfies the condition.

Example 4. Let us consider two facts $f_{1} = (v_{1}, e_{1}, v_{2})$ and $f_{2} = (v_{3}, e_{2}, v_{4})$. The not-informative likelihoods associated with each fact are as follows:

$f_{1}:$

:
$\operatorname{Pr}(\text{CCS} = \texttt{notinf}) = 0.1,\operatorname{Pr}(\text{GCI} = \texttt{notinf}) = 0.3$ ,

$f_{2}:$

:
$\operatorname{Pr}(\text{CCS} = \texttt{notinf}) = 0.6,\operatorname{Pr}(\text{GCI} = \texttt{notinf}) = 0.5$ .

The signature type likelihoods associated with each fact, and sorted in decreasing order of probability, are as follows:

$f_{1}:$

: Pr(oncogene) = 0.7, Pr(tsg) = 0.2, Pr(biomarker) = 0.1,

$f_{2}:$

: Pr(oncogene) = 0.5, Pr(tsg) = 0.4, Pr(biomarker) = 0.1.

Then, let us set the sufficiency threshold $\alpha \text{ to } 0.7$ and the consistency threshold $\beta \text{ to } 0.4$. In this scenario, both *f*_1_ and *f*_2_ pass the sufficiency check, as Pr(CCS = notinf) and Pr(GCI = notinf) are lower than *α* for both facts. On the other hand, only *f*_1_ passes the consistency check, since none of the signature type likelihoods of *f*_2_ are large enough to overcome the others by a margin of *β*. In this regard, for *f*_1_, we have $\operatorname{Pr}(\texttt{oncogene}) - \operatorname{Pr}(\texttt{tsg}) \gt \beta$, which makes $\texttt{oncogene}$ the candidate gene class for the fact. Conversely, for *f*_2_, we have $\operatorname{Pr}(\texttt{oncogene}) - \operatorname{Pr}(\texttt{tsg}) \lt \beta$, which provides no candidate gene class for the fact. Therefore, *f*_1_ is tagged as ‘reliable’ and *f*_2_ as ‘unreliable’.

The facts that pass both sufficiency and consistency checks are tagged as ‘reliable’ and used to populate the KB. Prior to population, the edges of ‘reliable’ facts are labeled through the tagging function $\sigma(\cdot)$ with the gene class having the highest likelihood. Note that we do not claim that gene classes are definitive. Rather, gene classes—and supporting sentences—should be treated as complementary, textual evidence that strengthens the hypotheses on the expected roles of genes in cancer obtained through experimental data.

## Active learning

The facts deemed as ‘unreliable’ by the reliability testing component (Module 5) are taken over by the active learning process, which ranks them by ascending reliability score and returns the top-*k* automatically annotated sentences to domain experts for annotation.

Definition 10. **reliability score**

Let $(v_{1}, e, v_{2})$ be a fact, $\{A_{i}\}_{i=1}^{l}$ a subset of the aspects associated with *e* and *S* the set of signature types. Then, by taking a specific value *a*_*ij*_ for each aspect *A*_*i*_ of the subset, the reliability score is computed as

(4) $$ \operatorname{rel}(e) = -\frac{\sum_{i=1}^{l}\operatorname{Pr}(a_{ij})}{l}\cdot \mathrm{H}(S), $$

where $\mathrm{H}(S)$ is the entropy of the signature set *S*, computed as

(5) $$ \mathrm{H}(S) = - \sum_{\texttt{type} \in S}\operatorname{Pr}(\sigma(e) = \texttt{type})\cdot \log\operatorname{Pr}(\sigma(e) = \texttt{type}). $$

In this work, we compute the reliability score by considering the subset of CCS and GCI aspects and by taking their not-informative values $\{\text{CCS} = \texttt{notinf}, \text{GCI} = \texttt{notinf}\}$. Once computed, CORE uses the reliability score to perform uncertainty sampling (65). In other words, CORE ranks ‘unreliable’ facts by ascending order of reliability score. Then, the top-*k* automatically annotated sentences associated with these facts are returned to domain experts for manual annotation (Module 2).

## Implementation and experiments

We use CORE to build a KB for gene expression–cancer associations. To this end, we conducted comprehensive experiments to quantify the extracted knowledge and evaluate the RE methods used to build the KB. In addition, we performed a KB reconstruction task against the state-of-the-art showing CORE effectiveness.

### Knowledge base creation

**Data processing.** We use different resources to build the KB, which increase with each subsequent iteration of the KB construction process. Table 3 reports statistics for the resources used to build each KB version. In the first iteration (KB0), we only consider manually annotated data coming from CoMAGC, OncoSearch and BioXpress. We revised these annotations to make them compliant with the annotation schema presented in Section (Manual annotation).

**Table 3.** Raw statistics for the KB versions. Rows represent the number of raw instances considered to build the KB.

		KB0	KB1	KB2	KB3
Manual	CoMAGC (revised)	821	821	821	821
	OncoSearch (revised)	157	157	157	157
	BioXpress (revised)	74	74	74	74
	DisGeNET (batch 1)	–	–	250	250
	DisGeNET (batch 2)	–	–	–	249
Automatic	DisGeNET (batch 1)	–	184,859	184,609	184,609
	DisGeNET (batch 2)	–	–	184,858	184,609
	PubMed (citing papers)	–	–	–	2,841,096
Total		1,052	185,911	370,769	3,211,865

Then, in the second iteration (KB1), we introduce DisGeNET data, on which the CORE system deploys the RE methods. DisGeNET collects data on different ‘coarse-grained’ gene-disease associations from several resources and covers most human diseases. Regarding gene expression–cancer associations, DisGeNET contains automatically extracted data that have been identified from the literature using text-mining techniques (28, 66, 67). For each gene–disease association, DisGeNET provides the publication(s) supporting the association, a representative sentence from each publication, the original source, as well as information on the gene and disease involved in the association. Thus, sentences within DisGeNET can be used as a high-quality starting point from which multi-aspect relationships can be extracted.

After the construction of KB1, the active learning process ranks ‘unreliable’ facts and returns the top-*k* sentences for manual annotation. This new set of manually annotated sentences—together with a second batch from DisGeNET—are added to previously used data and employed to build KB2. In the last iteration (KB3), we collect from PubMed the articles citing those stored within KB2. Then, the CORE system relies on the NERD component to extract gene and cancer entities from titles and abstract sentences and deploys RE methods on them. Finally, PubMed and top-*k* ‘unreliable’ sentences from KB2 are integrated into KB3 construction.

**Manual annotation.** The manual annotation process has been carried out by a clinical expert. The annotator has been given the target sentence to annotate/validate, together with the corresponding PubMed article from which it has been extracted—from either the title or the abstract.

**System parameters.** The parameters required by CORE are the sufficiency and consistency thresholds *α* and *β* and the number *k* of sentences to be returned for manual annotation during active learning. Sufficiency and consistency thresholds regulate the degree of reliability of the facts in the KB. A low sufficiency combined with a high consistency threshold leads to fewer facts but with a high level of reliability. Empirically, we set *α* = 0.7 and *β* = 0.4. We set *k* = 250, meaning that 250 sentences are reannotated after each iteration. Note that system parameters can be adjusted as the KB size increases.

**KB statistics.** From the statistics reported in Table 4, we draw some considerations. First, we can see that the ratio between the sentences stored in the KB and the input ones decreases at each iteration. From the first iteration, CORE uses 62% of the input sentences to build KB0, and we move to 52% to build KB1, 26% for KB2 and only 14% for KB3. Such a decrease reflects the use of reliability tests and active learning, which makes the system more selective and accurate. In particular, active learning leads the system to refine the RE methods at each iteration, thus reducing false positives as well as ‘unreliable’ facts as shown in Table 5, which presents the reduction statistics of ‘unreliable’ facts. We see that the number of facts deemed as ‘unreliable’ in one iteration decreases in the next ones, confirming the effectiveness of active learning.

**Table 4.** Partition, absolute and conditional statistics for KB.

		KB0	KB1	KB2	KB3
Partition	Manual	655	585	605	592
	Automatic	–	96 531	95 282	435 283
Absolute	Sentence	655	97 116	95 887	435 875
	Article	411	69 462	65 236	161 449
	Gene	329	9,483	9981	21 005
	Cancer	98	1479	1554	1665
	Fact	512	71 554	89 999	153 016
Conditional	Sentence/article	1.59	1.40	1.47	2.70
	Sentence/fact	1.28	1.67	1.56	3.10
	Article/fact	1.09	1.67	1.56	2.10

**Table 5.** Reduction statistics for unreliable facts. For each KB version (rows), we report the number of unreliable facts present in that version that are also found in subsequent versions (columns).

		KB0	KB1	KB2	KB3
Insufficient	KB0	10	5	5	5
	KB1	–	9055	2308	1135
	KB2	–	–	4515	2452
Inconsistent	KB0	22	18	15	17
	KB1	–	6135	3837	3704
	KB2	–	–	11 380	7786

Second, the large number of different genes and cancers in KB3 highlights the scalability of the approach. In this regard, KB3 contains 21 005 genes, which cover 70% of the 30 000 estimated genes in the human genome.^2^ On the other hand, through the integration of DisGeNET data, KBs 1–3 contain most of the (known) cancer types involved in gene expression–cancer associations. Together, this large number of genes and cancer types leads to more than 150 000 ‘reliable’ facts. Table 6 presents the distribution of these facts according to the corresponding signature type.

**Table 6.** Signature type statistics for each KB version.

Signature type	KB0	KB1	KB2	KB3
Biomarker	390	59,147	69,409	105,089
Oncogene	87	8,833	13,501	35,520
Tumor Suppressor Gene	35	3,574	7,089	12,407

Finally, KB3 represents one of the largest literature-derived KBs with fine-grained facts about gene expression–cancer associations. Compared to KB3, BioXpress and OncoMX—both relying on DEXTER text-mined results—contain less literature-derived data. Specifically, BioXpress integrates DEXTER gene expression–cancer associations for 2024 genes in lung cancer, 115 glycosyltransferases in 62 cancers and 826 microRNAs in 171 cancers (24). On the other hand, OncoMX integrates 22 904 gene expression–cancer associations between 5524 genes/microRNAs and 272 cancer types, extracted by DEXTER from 36 196 sentences in 25 860 PubMed articles. Although larger, OncoMX is still an order of magnitude smaller than KB3. Besides, both BioXpress and OncoMX only report CGE values between cancer and normal samples, thus providing less comprehensive information than CORE to model gene expression–cancer associations. A different situation occurs with OncoSearch, which contains 451 798 sentences expressing 7555 genes and 1717 cancer types, leading to 2295 oncogenes, 1549 tumor suppressor genes and 6779 biomarkers. Compared to OncoSearch, KB3 contains less sentences and cancer types. However, OncoSearch does not perform reliability tests and therefore ingests any annotated sentence. If we also consider the facts deemed as ‘unreliable’ by CORE when building KB3, then the number of sentences and cancer types becomes 1 037 845 and 1767, respectively. Thus, KB3 integrates a smaller number of sentences and cancer types to seek for a higher quality.

### Relation extraction evaluation

**Datasets.** We evaluate the effectiveness of the CGE, CCS and GCI extraction methods using three incremental sets of manually annotated data. Table 7 reports the statistics of these aspect extraction datasets. The first dataset (DS0) derives from the seed batch of manually annotated data used to build KB0. The second (DS1) and third (DS2) ones integrate additional data coming from the subsequent sets of 250 sentences returned by the active learning process. DS0 contains 1052 annotated sentences, which increased by 23% in DS1 and a further 19% in DS2.

**Table 7.** Statistics of the aspect extraction datasets. We provide the percentage increase from one version to the next

Aspect	Value	DS0	DS1	DS2
CGE	up	524	604 (+15%)	679 (+12%)
	down	219	263 (+20%)	311 (+18%)
	notinf	309	430 (+39%)	550 (+28%)
CCS	progression	605	719 (+19%)	829 (+15%)
	regression	134	147 (+10%)	162 (+10%)
	notinf	313	431 (+38%)	549 (+27%)
GCI	causality	189	227 (+20%)	266 (+17%)
	observation	548	634 (+16%)	719 (+13%)
	notinf	315	436 (+38%)	555 (+27%)
Total		1,052	1,297 (+23%)	1,540 (+19%)

Regarding the GCC extraction method, which serves as a sentence utility binary classifier, we use DisGeNET to create a large-scale semi-automatically annotated dataset. Similar to (68), we employ automatically extracted data from DisGeNET to build training and validation sets while relying on manually curated data for the test set. Table 8 reports the statistics for the sentence utility classifier dataset. For training and validation, DisGeNET sentences conveying a gene expression–cancer association were labeled as $\texttt{expression}$ and those conveying any other type of association as $\texttt{other}$. For test, DS2 sentences were used as $\texttt{expression}$ candidates and manually curated sentences from DisGeNET as $\texttt{other}$.

**Table 8.** Statistics of the sentence utility classifier dataset.

Class	Training	Validation	Test
expression	18,555	6,185	1,540
other	18,876	6,292	825

We create a unique dataset for the sentence utility classifier as the method is only applied to PubMed sentences during KB3 construction. PubMed is very general, and most of the sentences are not about gene expression–cancer associations, so the sentence utility classifier is critical for the CORE extraction process. Conversely, the sentence utility classifier is not needed on DisGeNET sentences because they are of high quality, and a filtering process has already taken place before their integration within it.

**Set-up.** For training, we set the batch size to 16 and the learning rate to 2e-5 with linear warm-up followed by linear decay (63), as suggested in (62). The CGE, CCS and GCI extraction methods perform multi-class classification and are trained using a standard cross entropy loss function. The sentence utility classifier performs binary classification and employs a binary cross-entropy loss.

We perform 10-fold cross-validation to evaluate CGE, CCS and GCI methods. For each iteration, we train the RE methods for 10 epochs, choose the best epoch on a validation set consisting of 25% of the training folds and report the corresponding results for the test fold. Instead, given the large size of the GCC extraction dataset, we train the sentence utility classifier for five epochs, pick the best epoch on the validation set and report the results on the test set.

**Results.** Table 9 reports the average performances of the CGE, CCS and GCI extraction methods on the different dataset versions.

**Table 9.** Aspect extraction performances

Dataset	Aspect	Accuracy	Precision	Recall	F1
DS0	CGE	0.8812	0.8870	0.8812	0.8792
	CCS	0.8593	0.8650	0.8593	0.8600
	GCI	0.8194	0.8305	0.8194	0.8212
DS1	CGE	0.8543	0.8574	0.8543	0.8526
	CCS	0.8404	0.8436	0.8404	0.8400
	GCI	0.8150	0.8269	0.8150	0.8142
DS2	CGE	0.8760	0.8813	0.8760	0.8746
	CCS	0.8481	0.8515	0.8481	0.8478
	GCI	0.8266	0.8314	0.8266	0.8259

We can see that all the three methods perform well on the task—above 0.80 for each measure—with peak performances on CGE and slightly lower performances on GCI. These results underline the differences between aspects, where CGE is most explicit in sentences—and therefore easier to extract—whereas GCI is less evident—and therefore more difficult to predict. CCS extraction is in between.

This experiment shows the effectiveness of the RE methods and their stability as they do not regress as the dataset size increases. In this regard, we recall that RE methods are retrained from scratch at each iteration and not fine-tuned with new data from the active learning process. Thus, such consistent performances across dataset versions highlight the robustness and reliability of the RE methods.

Regarding GCC extraction, the sentence utility classifier achieves an accuracy of 0.8825 and a precision, a recall and an F1 value of 0.8824, 0.8825 and 0.8803, respectively. The results highlight the viability of training coarse-grained RE methods using automatically annotated data from DisGeNET (68) and show the effectiveness of the trained method on a manual test set. Thus, the sentence utility classifier is reliable enough to be used as filter on new and heterogeneous sentences gathered from PubMed.

### Knowledge base reconstruction

**Setup.** We further evaluate the effectiveness of the CORE system on a KB reconstruction task, in which we hold out a portion of an existing KB with associated sentences and we assess CORE ability to recover it. To this end, we hold out from BioXpress the set of 9636 sentences annotated by DEXTER (SoTA for gene expression–cancer annotations), and we evaluate the CORE system on them. Note that such sentences are not part of those used to train the CORE RE methods. Given that BioXpress only reports CGE values between cancer and normal samples, we restrict our evaluation to CGE extraction. As a further experiment, we also apply DEXTER to DS2 to evaluate its ability to generalize to heterogeneous sentences, whose syntactic structure can differ from its predefined patterns.

**Results.** Table 10 reports the CORE system performance on the BioXpress reconstruction task after each (re-)training of the RE methods, as well as DEXTER performance on DS2. We can see that each CORE version consistently achieves performances above 0.95 for each measure. In particular, CORE1 improves over CORE0 by 2% and reaches a performance plateau, where CORE2 also stabilizes with an accuracy of 0.9706 and a precision, a recall and an F1 value equal to 0.9827, 0.9706 and 0.9766, respectively. The results show the effectiveness of the CORE system in recovering BioXpress using a limited amount of manual annotations to train the RE methods. On the other hand, the poor performance of DEXTER on DS2 highlights a lack of flexibility that hampers its applicability to heterogeneous sentences. To further support this intuition, we observe that for DEXTER, recall presents the worst performance (0.3256) if compared to precision (0.6034). This underlines DEXTER’s expert system nature based on pattern-matching, which, although precise, fails to generalize beyond its set of predefined patterns.

**Table 10.** CORE system performance on the BioXpress reconstruction task. We also report DEXTER performance on DS2.

Dataset	Method	Accuracy	Precision	Recall	F1
BioXpress	CORE0	0.9544	0.9601	0.9544	0.9572
	CORE1	0.9703	0.9831	0.9703	0.9766
	CORE2	0.9706	0.9827	0.9706	0.9766
DS2	DEXTER	0.3256	0.6034	0.3256	0.2882

## Knowledge base exploration

We perform some exploratory queries to analyse the contents of the largest KB produced by CORE, that is KB3. The SPARQL queries used to explore KB3 can be found in Appendix A.

### Genes most involved in cancer diseases

**Figure 3.**
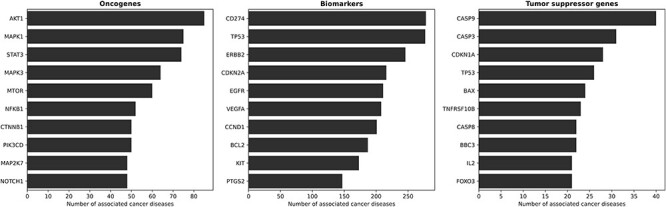
The ten most involved genes (and their roles) in cancer diseases. From left to right, the figures present the ten most involved oncogenes, biomarkers and tumor suppressor genes, respectively. AKT1 is the most prominent oncogene, with wide expression in various tissues. Other known oncogenes include MAPK1, MAPK3 and STAT3. Proto-oncogenes such as ERBB2, EGFR and BCL2 show altered expression levels in cancer, but lack sufficient evidence to be identified as oncogenes, thus fitting our definition of biomarkers. TP53 represents an interesting case, as it functions as a biomarker and a tumor suppressor gene for several diseases, with its classification evolving over time.

Figure 3 illustrates the top ten oncogenes, biomarkers and tumor suppressor genes associated with cancer. Among the oncogenes, AKT1 emerges as the predominant gene implicated in cancer diseases within KB3. AKT1 exhibits widespread expression in various tissues (69, 70). Other known oncogenes are MAPK1 and MAPK3, frequently involved in oncogenesis, tumor progression and drug resistance (71) and STAT3 (72). Regarding biomarkers, there are several known proto-oncogenes such as ERBB2 (73), EGFR (74) and BCL2 (75). Proto-oncogenes fit our definition of biomarkers, i.e. genes that show altered expression levels in cancer but do not (yet) have enough evidence to be identified as oncogenes or tumor suppressor genes. A different situation occurs with TP53, which presents an interesting scenario as it is a biomarker and a tumor suppressor gene for many diseases. Over the years, the scientific understanding of TP53 has evolved, initially classifying it as an oncogene (76), then recognizing it as a tumor suppressor (77) and, more recently, under certain conditions, acknowledging its re-emergence as an oncogene (78). Thus, thanks to its probabilistic, fact-centric and evidence-based approach, the CORE system can capture such a dynamic scenario—which is proper for scientific discourse.

### Most discussed genes, cancer diseases and facts

**Figure 4.**
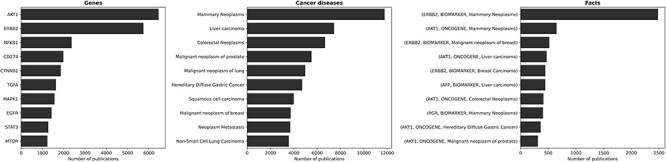
The ten most discussed genes, cancer diseases, and facts within the literature. The most discussed genes are those most involved in cancer diseases, with a focus on breast, colorectal, prostate, and lung cancer—i.e., the most common cancer types worldwide. Consequently, the most discussed facts refer to gene expression-cancer associations involving these specific genes and diseases.

Figure 4 presents the genes, diseases and facts that have garnered the most attention in the scientific literature. Naturally, the most discussed genes align with the ones most involved in cancer diseases. The most discussed topics concerning cancer predominantly revolve around breast, colorectal, prostate and lung cancer types. This outcome is fitting, as these cancer types are the four most common cancer types worldwide.^3^ As a consequence, the most discussed facts pertain to gene expression–cancer associations involving the aforementioned genes and diseases.

### Longest-discussed fact in the literature

**Figure 5.**
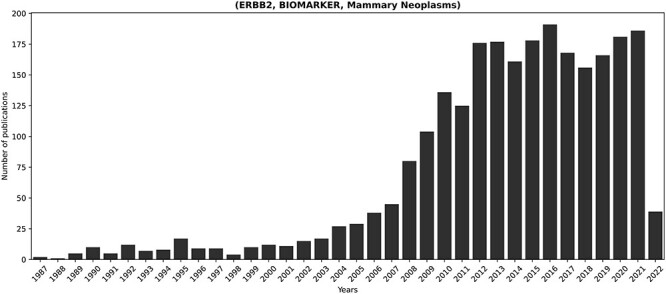
Temporal progression of publications concerning the longest-discussed fact in literature: (ERBB2, BIOMARKER, Mammary Neoplasms). ERBB2 is a known proto-oncogene, amplified or overexpressed in around 30% of human breast cancers (73). Its relevance in breast cancer justifies the prominent presence of the corresponding fact in the scientific discourse.

Figure 5 showcases the temporal progression of publications concerning the fact most extensively discussed in KB3 (i.e. ERBB2, BIOMARKER, mammary neoplasms). ERBB2 is a known proto-oncogene that plays an important role in human malignancies and is amplified or overexpressed in 30% of human breast cancers (73). Therefore, the relevance of ERBB2 in breast cancer well motivates its prominence within the scientific discourse.

## Search platform

**Figure 6.**
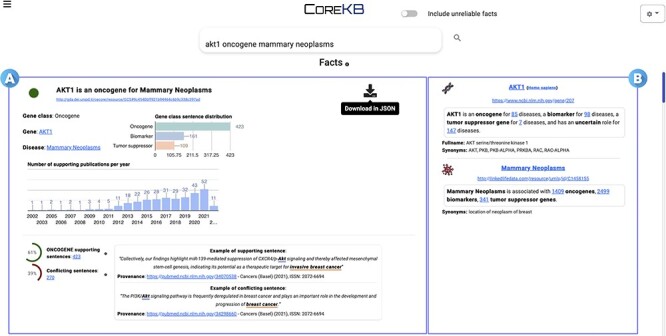
COREKB Search Engine Result Page first result for the query ‘AKT1 oncogene mammary neoplasms’. The retrieved facts are organized as cards providing several information concerning (A) the gene, cancer and their relationship and (B) specific information concerning the entities—i.e. gene and the related cancer expression—involved in the association. In addition, card (A) includes infometrics and bibliometrics information to provide further insights. The contents of the cards are available for download in JSON format through the dedicated download button.

The KB generated by CORE can also be accessed via COREKB (30), an intuitive and easy-to-use search platform for searching scientific facts over gene expression–cancer associations. COREKB allows users to search for gene–cancer associations and entities using free-text or structured search queries. The interface provides several features, including autocomplete facilities, entity cards summarizing the major gene–cancer relationships and entity landing pages, and users can easily switch between free-text and structured search interfaces. The system also offers a simple toggle button to include/exclude unreliable facts from the search results. The search results are presented as a list of cards showing the information concerning the scientific facts matching the user-provided query. Card information can also be downloaded in JSON format via the dedicated download button. Figure 6 shows the first result of the Search Engine Result Page for the query ‘AKT1 oncogene mammary neoplasms’.

### Architecture

COREKB’s architecture consists of multiple components synergically cooperating to facilitate the search and retrieval of scientific facts—i.e. gene expression–cancer associations supported by the scientific literature. The architecture includes a web-based front–end interface built with React.js and a back-end for the business logic, Representational State Transfer Application Programming Interfaces and services built with the Python web framework Django. The system relies on a PostgreSQL database coupled with a Virtuoso Resource Description Framework triple store to memorize the KB. Moreover, Redis is exploited as an efficient in-memory data store and access broker. A search and retrieval component implemented in Python performs NERD on the user-provided queries to identify entity mentions and, in turn, perform a structured search on the database. To this aim, a Redis in-memory dictionary of entities is exploited for fast entity identification.

Specifically, when a user query is received, the system assigns a score to each entity based either on an exact match (if it occurs) or on the number of matching terms in the case of a partial match. The score is normalized based on the entity’s length to avoid favoring longer entities at the expense of shorter ones. Then, the retrieved facts are ordered according to their scientific evidence support. In the case of multiple recognized entities, the system promotes gene–cancer pairs with the most matching associations.

### Interface

The interface reports the search results by organizing them into cards; it provides information such as gene class, symbol, cancer label, supporting and conflicting sentences, associated publications, gene class distribution and bibliometrics. The fact claim is emphasized using boldface and a colored circle, indicating the informativeness and reliability of the fact—i.e. green, red and gray colors, respectively, for reliable, unreliable and non-informative facts. Moreover, the interface includes links and references to related entries in external platforms like NCBI and Linked Life Data.^4^

For each gene or cancer entity, there is a dedicated landing page that displays comprehensive information. The landing page consists of two major cards. The first card presents detailed information about the entity, for instance, in the case of a gene, it shows its symbol, full name, type, synonyms, designations, last modified date, summary and gene class distribution for different cancer diseases. Long textual information can be expanded or collapsed on click for space-saving purposes. Instead, a second card shows the sentences involving the entity of interest, presented in a tabular form with filtering and sorting features. Users can resize columns, hover over sentences for getting information via tooltips or click on sentences for a separate pop-up view.

## Conclusions and future work

In this work, we presented CORE, a KBC system based on the combination of automated ML-based methods and domain experts. CORE presents a seamless, transparent and modular architecture that can be easily modified and where different components can be replaced without affecting the others. Among its main features, the reliability tests and the active learning process make the system suited to iterative KB versioning. That is, CORE performs iterative tests that measure the reliability of the extracted data and return small, selected samples to domain experts for annotation. The high-quality data generated through active learning are then used to reinforce CORE subsequent versions. We used CORE to build one of the largest literature-derived KBs containing fine-grained facts about gene expression–cancer associations. To show the robustness of the approach, we conducted extensive experiments that highlighted the ability of CORE to scale to large collections of heterogeneous data with limited human annotations. The KB generated by CORE can be accessed via a SPARQL endpoint (http://w3id.org/corekb/sparql) or through the COREKB search platform (https://gda.dei.unipd.it).

The CORE system is an ongoing effort carried out in partnership with medical centers. The expertise and insights of clinicians have been instrumental in developing a robust KBC system. Future work aims to improve the system by integrating advanced large language models (LLMs) as input sources. Robust validation mechanisms and collaboration with experts will be crucial to identify and ingesting reliable content generated by LLMs.

## Footnotes

1
https://gco.iarc.fr/tomorrow/en/dataviz/bubbles?sexes=0&mode=population

2
https://www.genome.gov/human-genome-project/

3
https://www.wcrf.org/cancer-trends/worldwide-cancer-data/

4
http://linkedlifedata.com

## Appendix SPARQL queries

Queries A1–A3 can be used to find the most involved genes (and their roles) in cancer diseases. Queries A4–A6 can be used to identify the most discussed genes, cancer diseases and facts. Query A7 can be used to find out the fact most extensively discussed in the literature. The code to cut and paste the queries in the SPARQL endpoint without syntax issues is available here: https://github.com/GDAMining/core/blob/main/sparql/example-queries.txt.

Querie A1. Find the ten most involved oncogenes in cancer diseases.

                **PREFIX** xsd: <http://www.w3.org/2001/XMLSchema#>
**PREFIX** core: <http://gda.dei.unipd.it/cecore/
             ontology/>
**SELECT** ?gene (**COUNT**(?fact) **AS** ?numFacts)
**WHERE**
{
   ?fact core: expressedBy ?gene;
         core: hasType “ONCOGENE”^^^^xsd:
               string.
}
**GROUP BY** ?gene
**ORDER BY DESC**(?numFacts)
**LIMIT** 10

Querie A2. Find the ten most involved biomarkers in cancer diseases.

                **PREFIX** xsd: <http://www.w3.org/2001/XMLSchema#>
**PREFIX** core: <http://gda.dei.unipd.it/cecore/
             ontology/>
**SELECT** ?gene (**COUNT**(?fact) **AS** ?numFacts)
**WHERE**
{
   ?fact core: expressedBy ?gene;
         core: hasType “BIOMARKER”^^^^ xsd:
               string.
}
**GROUP BY** ?gene
**ORDER BY DESC**(?numFacts)
**LIMIT** 10

Querie A3. Find the ten most involved tumor suppressor genes in cancer diseases.

                **PREFIX** xsd: <http://www.w3.org/2001/XMLSchema#>
**PREFIX** core: <http://gda.dei.unipd.it/cecore/
             ontology/>
**SELECT** ?gene (**COUNT**(?fact) **AS** ?numFacts)
**WHERE**
{
   ?fact core: expressedBy ?gene;
         core: hasType “TSG”^^^^ xsd:string.
}
**GROUP BY** ?gene
**ORDER BY DESC**(?numFacts)
**LIMIT** 10

Querie A4. Find the ten most discussed genes in the literature.

                **PREFIX** core: <http://gda.dei.unipd.it/cecore/
             ontology/>
**SELECT** ?gene (**COUNT**(**DISTINCT** ?article) **AS**
       ?numArticles)
**WHERE**
{
   ?fact core: expressedBy ?gene;
         core: supportedBy ?evidence.
 
   ?evidence core: extractedFrom ?article.
}
**GROUP BY** ?gene
**ORDER BY DESC**(?numArticles)
**LIMIT** 10

Querie A5. Find the ten most discussed cancer diseases in the literature.

                **PREFIX** core: <http://gda.dei.unipd.it/cecore/
             ontology/>
**PREFIX** umls: <http://linkedlifedata.com/
             resource/umls/id/>
 
**SELECT** ?disease (**COUNT**(**DISTINCT** ?article) **AS**
       ?numArticles)
**WHERE**
{
   ?fact core: involves ?disease;
         core: supportedBy ?evidence.
 
   ?evidence core: extractedFrom ?article.
   FILTER (?disease NOT IN (umls:C0086692,
           umls:C0027651, umls:C0006826,
           umls:C1306459))
}
**GROUP BY** ?disease
**ORDER BY DESC**(?numArticles)
**LIMIT** 10

Querie A6. Find the ten most discussed facts in the literature.

                **PREFIX** core: <http://gda.dei.unipd.it/cecore/
             ontology/>
**PREFIX** umls: <http://linkedlifedata.com/
             resource/umls/id/>
**SELECT** ?disease (**COUNT**(**DISTINCT** ?article) **AS**
       ?numArticles)
**WHERE**
{
   ?fact core: involves ?disease;
         core: supportedBy ?evidence.
 
   ?evidence core: extractedFrom ?article.
   FILTER (?disease **NOT IN** (umls:C0086692,
           umls:C0027651, umls:C0006826,
           umls:C1306459))
}
**GROUP BY** ?disease
**ORDER BY DESC**(?numArticles)
**LIMIT** 10

Querie A7. Find the longest-discussed fact in the literature.

                **PREFIX** core: <http://gda.dei.unipd.it/cecore/
             ontology/>
**SELECT** ?gene ?relation ?disease ?pubDate
       (**COUNT**(**DISTINCT** ?article) **AS**
       ?numArticles)
**WHERE**
{
   ?fact core: expressedBy ?gene;
         core: hasType ?relation;
         core: involves ?disease;
         core: supportedBy ?evidence.
 
   ?evidence core: extractedFrom ?article.
   ?article core: publicationYear ?pubDate.
 
   {
     **SELECT** ?fact (**COUNT**(**DISTINCT** ?pubDate) **AS**
            ?numYears)
     **WHERE**
     {
     ?fact core: supportedBy ?evidence.
      
     ?evidence core: extractedFrom ?article.
     ?article core: publicationYear ?pubDate.
     }
     **GROUP BY** ?fact
     **ORDER BY DESC**(?numYears)
     **LIMIT** 1
   }
}
**GROUP BY** ?gene ?relation ?disease ?pubDate
**ORDER BY ASC**(?pubDate)
